# Transforming endoscopic retrograde cholangiopancreatography: the role of artificial intelligence in pre-operative planning, intraoperative navigation, and post-operative risk prediction

**DOI:** 10.3389/fmed.2026.1879402

**Published:** 2026-07-22

**Authors:** Yaoqi Wang, Ya Peng, Zheng Wang, Peng Liu, Zhiyuan Chen

**Affiliations:** 1 Department of Gastroenterology, Hunan Provincial People's Hospital, The First Affiliated Hospital of Hunan Normal University, Changsha, Hunan, China; 2 Changsha Endoscopic Diagnosis and Treatment Technology Innovation Research Center for Biliary and Pancreatic Diseases, Changsha, China; 3 School of Computer Science, Hunan First Normal University, Changsha, China

**Keywords:** artificial intelligence, endoscopic retrograde cholangiopancreatography, intraoperative navigation, postoperative risk prediction, preoperative planning

## Abstract

Endoscopic Retrograde Cholangiopancreatography (ERCP) is a technically demanding procedure for pancreaticobiliary diseases. Artificial intelligence (AI) is transforming ERCP by improving diagnostic accuracy, procedural safety, and postoperative risk prediction. This review synthesizes AI applications across the entire workflow: preoperative planning using multimodal risk models, intraoperative navigation via real-time anatomical localization and augmented reality, and postoperative complication prediction with dynamic algorithms. AI also optimizes anesthesia and ambulatory surgery workflows. Challenges remain in data standardization, legal accountability, and clinical integration. We advocate for multicenter collaboration, ethical oversight, and comprehensive patient management systems to establish AI-assisted ERCP as a new standard for personalized interventional care.

## 1 Introduction

Endoscopic Retrograde Cholangiopancreatography (ERCP) is an indispensable modality for diagnosing and treating pancreaticobiliary diseases such as bile duct stones, strictures, and tumors. Despite its clinical utility, ERCP faces several challenges, including limited diagnostic accuracy, procedural complexity, considerable complication risks, and inefficiencies in data interpretation. Recent advances in artificial intelligence (AI) present transformative opportunities to enhance the precision and safety of ERCP (1–3). However, existing reviews have yet to systematically synthesize AI's role throughout the entire ERCP workflow. For instance, Osagiede's review highlights fragmented AI applications but lacks a comprehensive integration of preoperative, intraoperative, and postoperative risk prediction innovations (4). Similarly, Cao's review acknowledges the absence of a unified framework for AI-enhanced full-cycle management (5), hindering clinical translation and standardization.

**Figure 1:**
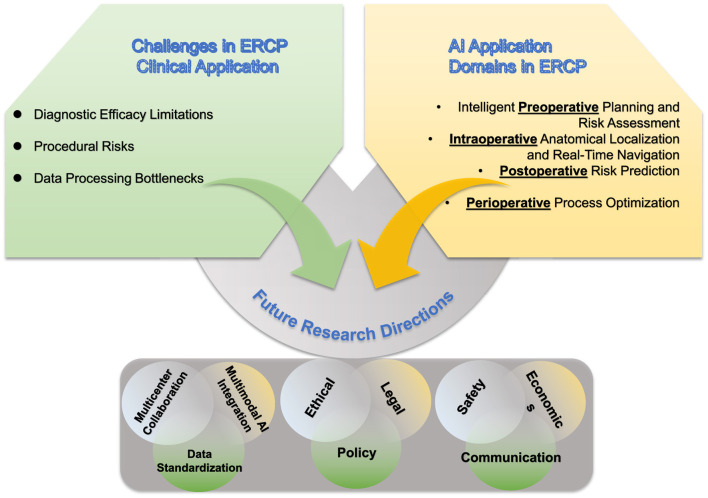
Graphical abstract: AI Enhanced Innovations in ERCP. This schematic summarizes the core themes of the review: (Top) Challenges in ERCP clinical application, including diagnostic efficacy limitations, procedural risks, and data processing bottlenecks. (Middle) Key AI application domains spanning preoperative planning, intraoperative navigation, postoperative risk prediction, and perioperative process optimization. (Bottom) Future research directions emphasizing multicenter collaboration, multimodal AI integration, ethical-legal-policy frameworks, safety-economics-communication considerations, and data standardization. AI, artificial intelligence; ERCP, endoscopic retrograde cholangiopancreatography.

Unlike prior reviews focusing on isolated techniques, this study establishes a comprehensive, system-wide framework for AI integration in ERCP. It critically examines cutting-edge implementations across two key dimensions: (1) establishing a methodological framework for deep AI-ERCP integration; and (2) analyzing AI applications in preoperative planning, intraoperative navigation, and postoperative risk prediction. This approach aims to provide essential references for medical AI standardization. Advancing multicenter validation and technological refinement will be pivotal in positioning AI-assisted ERCP as a tool to revolutionize pancreaticobiliary disorder management. To visually summarize the key themes and structure of this review, a graphical abstract has been constructed (Figure 1).

## 2 . Literature search and selection

### 2.1 Search databases

To ensure comprehensive coverage and authoritative evidence, the following databases were systematically searched:

English Databases: PubMed, Embase, Web of Science, IEEE Xplore, Cochrane Library.

Chinese Databases: CNKI, Wanfang Database, VIP Database.

### 2.2 Search strategy

The following search string was adapted for each database:

(“Endoscopic Retrograde Cholangiopancreatography” OR “ERCP”) AND (“artificial intelligence” OR “deep learning” OR “machine learning” OR “neural network”) AND (“preoperative planning” OR “risk assessment” OR “intraoperative navigation” OR “postoperative complications” OR “pancreatitis” OR “cholecystitis” OR “anesthesia”)

Date restriction: January 1, 2020–March 31, 2026.

### 2.3 Inclusion and exclusion criteria

Inclusion: (1) Original research developing or validating an AI/deep learning model for ERCP; (2) published 2020–2026; (3) clinical outcomes reported; (4) sample size ≥ 20 patients; (5) internal or external validation.

Exclusion: (1) Non-clinical studies; (2) duplicate datasets; (3) case reports/editorials; (4) no performance metrics.

Study selection process:

(1) Records identified (after deduplication): *n* = 847.
(2) Title/abstract screening: *n* = 533 (excluded *n* = 314).
(3) Full-text assessment: *n* =276 (excluded *n* = 214).
(4) Studies included in synthesis: *n* = 62.

## 3 . Challenges in ERCP clinical application

### 3.1 Diagnostic efficacy limitations

The diagnostic accuracy of ERCP is hindered by technical limitations, variability in operator expertise, and anatomical heterogeneity. Although adjunctive brush cytology or tissue biopsy achieves specificity up to 0.99 for differentiating benign from malignant strictures, it has a sensitivity below 0.50 (6). Core limitations that critically reduce efficacy include blind spots in subtle lesion detection, interference from overlapping imaging artifacts, and insufficient temporal resolution in dynamic contrast imaging.

### 3.2 Procedural risks

ERCP is a technically demanding endoscopic procedure characterized by a steep learning curve. Studies show novice endoscopists have a 3.2-fold higher rate of biliary cannulation failure compared to experts, while experienced operators still encounter a failure rate between 18% and 22% (7). Each additional cannulation attempt increases the risk of post-ERCP pancreatitis (PEP) by 35%, with the overall risk reaching 15.7% when pancreatic duct opacification occurs (7, 8). Furthermore, the procedure carries significant risks of bleeding (1.3% to 6.0%) and perforation (0.1% to 0.6%), which can adversely affect both short-term recovery and long-term outcomes (9). These challenges are exacerbated by variable operator skill levels and inconsistent adherence to standardized protocols, often leading to anatomical oversights (10, 11).

### 3.3 Data processing bottlenecks

Conventional ERCP procedures generate between 50 and 100 low-resolution frames, which limits timely image interpretation, quantitative bile flow assessment, and systematic documentation of pancreatic duct opacification patterns. Critically, variations in image quality and lesion annotation across institutions hinder multicenter studies and reduce the generalizability of AI models.

ERCP faces multiple challenges related to diagnosis accuracy, procedural safety, and data processing efficiency in the management of biliary and pancreatic diseases. These limitations compromise clinical decision-making, increase medical risks, and lead to inefficient use of healthcare resources. Addressing these challenges necessitates integrated solutions that combine technological innovation —particularly AI-driven diagnostics—with process optimization through standardized quality frameworks.

## 4 AI application domains in ERCP

### 4.1 Intelligent preoperative planning and risk assessment

**Figure 2:**
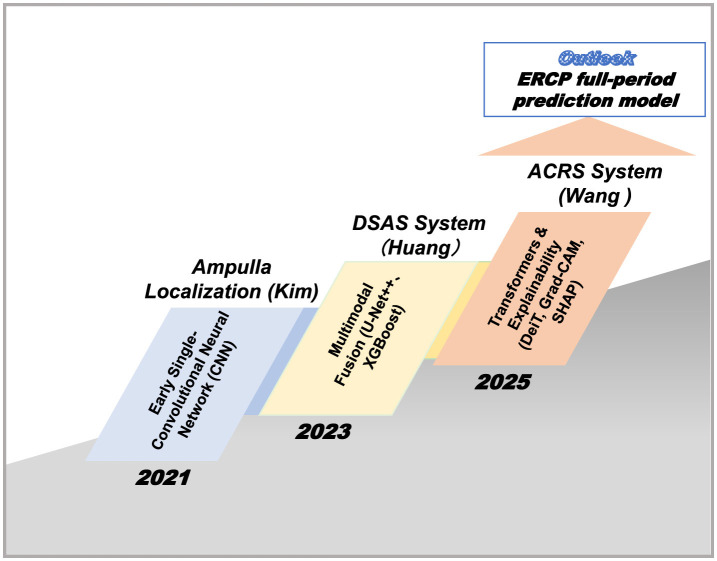
Evolutionary Progression of AI Models in ERCP. This figure illustrates the stepwise advancement from pioneering single-modal CNNs to integrated multimodal fusion systems and transformer-based explainable AI. This progression culminates in a future vision of comprehensive full-cycle prediction models spanning preoperative, intraoperative, and postoperative ERCP management, enabling unified clinical decision support. ACRS, assessment of stone removal challenges; AI, artificial intelligence; CNN, convolutional neural networks; DSAS, difficulty scoring system; DeiT, data-efficient image transformers; ERCP, endoscopic retrograde cholangiopancreatography; Grad-CAM, gradient-weighted class activation mapping; SHAP, shapley additive explanations; XGBoost, eXtreme gradient boosting.

Precise patient selection and risk stratification are crucial for optimizing ERCP outcomes. Recent AI advances enable multimodal systems, transforming the management of pancreaticobiliary disorders. Hou et al. (12)'s EfficientNet-B5 model integrates 27 non-invasive parameters—including serum amylase, bilirubin levels, and ultrasound findings—to identify high-risk candidates, demonstrating a 2.5-fold improvement in accuracy over manual interpretation for detecting bile duct dilation >10 mm in multicenter validations. Huang et al. (13)'s Difficulty Scoring System (DSAS) uses U-Net++ for 3D common bile duct (CBD) contour and stone segmentation, enabling automated calculation of the stone-to-duct ratio and a four-tier scoring system with superior concordance to expert assessment (13, 14). Our ACRS system combines endoscopic imaging (Dei T Data-efficient Image Transformers) with clinical data analyzed via XGBoost (eXtreme Gradient Boosting), incorporating explainability methods such as gradient-weighted class activation mapping (Grad-CAM) and Shapley Additive explanations (SHAP), achieving 100% sensitivity and 83% accuracy in surgical planning (15–18). Collectively, these developments mark a major evolution toward AI-driven preoperative evaluation. AI evolution in preoperative planning can be divided into three phases (Figure 2): from early unimodal convolutional neural networks (CNNs) to current models incorporating Transformer architectures and explainability techniques (Grad-CAM, SHAP). This progression reveals several trends: (1) Unimodal to multidimensional: Expansion from purely image-based analysis to multimodal incorporation of clinical and biochemical data; (2) Static to dynamic: Transition from static CNN image processing to 3D spatiotemporal networks capable of handling sequential data; (3) Opaque to interpretable: Adoption of explainable AI (XAI) methods that balance high performance with clinical interpretability and trust.

### 4.2 Intraoperative anatomical localization and real-time navigation

Ampullary anatomical variations remain a primary cause of difficult ERCP cannulation, underscoring the need for precise anatomical identification. While Kim et al. (19)'s CNN-based localization system achieves expert-level real-time anatomical assessment, it encounters critical translational challenges: its recall rate for difficult cannulations is only 61.1%, despite demonstrating high efficacy in routine cases. Key limitations include: (1) an overreliance on positional data while neglecting morphological determinants, such as orifice orientation, fold patterns, which account for approximately 42% of cannulation difficulties; (2) the exclusion of dynamic intraoperative variables such as peristalsis and hemorrhage, compromising predictive accuracy when anatomical localization succeeds but cannulation fails. Recent advancements in intelligent navigation systems address these gaps by leveraging multimodal imaging and real-time visualization (20). Pioneered by Dr. Li's team at Wuhan University, the biliary-pancreatic EUS navigation system enables intelligent visualization of guideline-recommended anatomy landmarks (21, 22). This system evolved into the deep learning-based EUS Automatic Image Reporting System (EUS-AIRS), integrating intelligent anatomical localization, automated pathology analysis, and real-time structured reporting (23). Clinical validation confirms EUS-AIRS' superiority: it outperformed expert endoscopists in assessing biliopancreatic structural integrity (90.8% vs. 70.5%) and excels in standardized image capture accuracy and completeness. Building on these innovations, next-generation ERCP navigation systems are emerging. Future architectures aim to transcend the limitations of CNNs via 3D spatiotemporal networks dynamically correlating multi-angle sequence imaging with procedural difficulty. The integration of real-time navigation, intelligent decision support, and automated reporting will culminate in comprehensive AI-driven systems. These “digital navigator” platforms are expected to utilize augmented reality to guide critical steps, such as duct cannulation and stent placement, thereby heralding a paradigm shift toward intelligent diagnosis and therapy in interventional gastroenterology.

While the EUS-AIRS platform was originally validated for linear EUS, its core architectural principles—intelligent anatomical localization, automated pathology annotation, and real-time structured reporting—are directly transferable to ERCP. Specifically, the deep learning models for bile duct segmentation and station recognition can be retrained on ERCP fluoroscopic sequences after adjustment for contrast dynamics and catheter artifacts. However, three adaptations are necessary for ERCP navigation: (1) conversion from 2D static image analysis to 3D spatiotemporal reconstruction using recurrent or Transformer-based architectures; (2) integration of real-time cannulation force feedback (not available in EUS); and (3) validation against ERCP-specific outcomes (e.g., selective cannulation success). Preliminary ERCP-directed systems, such as Kim et al. (19)'s CNN for ampulla detection, have achieved 84.5% accuracy, but full translation of the EUS-AIRS framework into a dedicated ERCP “digital navigator” remains an ongoing effort.

### 4.3 Postoperative complication prediction and management

#### 4.3.1 Precision prediction and management of post-ERCP pancreatitis

PEP is a major complication associated with prolonged hospitalization and potentially life-threatening outcomes, underscoring the urgent need for predictive tools that surpass current prophylactic measures such as Non-Steroidal Anti-Inflammatory Drugs (NSAIDs) and stenting (24). Conventional risk scoring based primarily on demographic factors fails to provide individualized stratification (25). In contrast, Meng et al. (26) developed an 8-parameter predictive model—including factors such as sex, age, and duct cannulation—derived from 3,021 Canadian ERCP cases, achieving 79% predictive accuracy and outperforming traditional systems (27). Their multimodal approach integrates preoperative blood tests, ERCP imaging data, and procedural factors (e.g., cannulation attempts, contrast volume), enabling dynamic risk assessment across three phases: preoperative baseline, intraoperative real-time difficulty assessment, and early postoperative laboratory evaluation. This approach achieved 89% predictive accuracy in multicenter validation (28). A Network meta-analysis further confirmed that AI-guided risk stratification enables personalized prevention strategies—such as combined indomethacin and stenting for high-risk patients—resulting in significant reductions in PEP incidence (29). Collectively, these AI-driven methodologies facilitate comprehensive data synthesis for precise PEP prediction and proactive management. Despite existing implementation barriers, such AI-driven assessments show strong potential for incorporation into standardized preoperative ERCP protocol (30).

#### 4.3.2 Prediction and management of post-ERCP cholecystitis

Post-ERCP cholecystitis (PEC) is a serious but often overlooked complication, particularly in patients with an intact gallbladder and common bile duct stones. Its reported incidence ranges from 0.5% to 9.4% depending on the study population and observation window, and delayed recognition may necessitate emergency cholecystectomy or percutaneous drainage (31, 32). However, reliable preoperative risk stratification has remained elusive due to the complex interplay among patient characteristics, gallbladder status, and procedural variables. Addressing this gap, Zhang et al. (33) developed a machine learning model using a random forest (RF) algorithm to predict PEC in a large cohort of 1,117 patients with common bile duct stones and gallbladder in-situ (90 PEC events, incidence 8.06%). Unlike conventional logistic regression, the RF model identified six top-ranked predictors: white blood cell count, endoscopic papillary balloon dilatation (EPBD), postoperative increase in white blood cell count, residual common bile duct stones after ERCP, serum amylase levels, and mechanical lithotripsy. The model achieved a sensitivity of 0.822, specificity of 0.853, accuracy of 0.855, and area under the curve (AUC) of 0.890, significantly outperforming the logistic regression model (AUC 0.864). External validation on an independent cohort of 117 patients (11 PEC, 9.40%) confirmed robust performance (AUC 0.889). For clinical translation, an open-access online prediction platform was established, allowing endoscopists to input six readily available parameters and obtain individualized PEC risk estimates.

The superiority of the RF model over traditional regression stems from its ability to handle non-linear interactions and high-dimensional clinical data without overfitting. Notably, several identified risk factors—such as EPBD and mechanical lithotripsy—are modifiable procedural variables, offering opportunities for preventive strategies. For instance, in patients with elevated predicted PEC risk, operators might consider minimizing balloon dilatation, ensuring complete stone clearance, or administering prophylactic antibiotics (34). Integrating this predictive model into perioperative workflows enables multimodal data fusion, thereby optimizing clinical decision-making and advancing precision medicine in ERCP management. Nevertheless, current evidence remains limited to single-center retrospective data, and prospective multicenter validation is urgently needed to confirm generalizability. Future efforts should also focus on embedding PEC prediction into a unified perioperative risk dashboard alongside post-ERCP pancreatitis and other complications, forming a comprehensive AI-enhanced safety protocol for ERCP.

### 4.4 Perioperative process optimization

#### 4.4.1 Intelligent management in anesthesiology

Artificial intelligence is revolutionizing anesthesiology across several domains: (1) airway management, (2) ultrasound diagnostics, (3) smart drug delivery, (4) intraoperative monitoring, and (5) critical care applications. These advancements optimize clinical workflows and improve patient outcomes (35). In the context of ERCP anesthesia, AI-assisted airway management demonstrates distinct advantages, exemplified by the University of Zurich's REALITI intubation system (36). This system enables real-time detection of laryngeal anatomical landmarks, annotates recognized regions using augmented reality (AR) technology, and guides the operator to switch to autonomous mode. Upon endoscopic confirmation of glottic positioning, it generates 3D tracheal reconstructions for operator verification. Endotracheal intubation, while ensuring airway security, is associated with an increased risk of postoperative cognitive dysfunction (POCD) and prolonged recovery times (mean delay: 32 min). AI technologies address these limitations through several advancements: (1) a system incorporating 387 variables for predicting multiple complications (e.g., pneumonia, acute kidney injury); although its clinical applicability is currently restricted by the challenge of collecting such extensive data, future efforts should focus on feature selection and dimensionality reduction; (2) models integrating 23 modifiable perioperative factors (e.g., fluid management, early mobilization) to generate dynamic, targeted interventions such as optimized fluid protocols and stepwise analgesia regimens (37, 38).

Moreover, AI has been applied to predict intraprocedural hypoxaemia during ERCP under monitored anesthesia care (MAC). Kang et al. (39) developed a gradient boosting machine (GBM) ensemble model using 16 preprocedural variables (including age, BMI, and habitual snoring), achieving an AUC of 0.7336 and outperforming conventional logistic regression; the model identified a high-risk group with a 13.14% hypoxaemia rate vs. 3.03% in the low-risk group, enabling anesthesiologists to consider general endotracheal anesthesia for high-risk patients instead of MAC (40). Furthermore, AI-based tools have been explored for perioperative medication management. Malik et al. (41) evaluated ChatGPT-4 with retrieval-augmented generation (RAG) for anticoagulation guidance prior to ERCP; the model achieved 75% full accuracy for guideline-consistent recommendations, and 90% of surveyed gastroenterologists believed such AI tools could reduce medication errors, though concerns over data confidentiality and the need for human oversight remained.

#### 4.4.2 Ambulatory surgery management

The shift toward ambulatory (day-case) ERCP emphasizes the need for highly efficient, standardized, and safe perioperative workflows. While dedicated AI guidelines for ambulatory ERCP are not yet established, recent expert consensus and management platform developments provide a critical foundation for AI integration. The Chinese Expert Consensus on the Management of ERCP Day Surgery (2025 Edition) outlines a standardized framework for patient selection, perioperative protocols, and discharge criteria, aiming to complete high-quality admission-to-discharge cycles within 24 h (42). This framework inherently creates the structured data environment and process definitions necessary for AI deployment.

Concurrently, the construction of intelligent management platforms for daytime surgery demonstrates how AI and digital tools can be operationalized within such frameworks (43). These platforms typically integrate several AI-driven modules: (1) intelligent patient selection and risk assessment using predictive models, (2) ML-optimized scheduling that aligns procedural slots with case complexity, (3) perioperative decision support, and (4) automated follow-up and monitoring systems. By fusing data from electronic health records, real-time physiological monitors, and post-discharge patient-reported outcomes, these platforms facilitate a closed-loop, precision management system.

Therefore, the current trajectory involves applying existing AI technologies—such as risk prediction, workflow optimization, and automated reporting—within the structured pathway defined by the latest ambulatory ERCP consensus. This synergy between evidence-based clinical guidelines and intelligent digital platforms is establishing a new benchmark for efficient, safe, and patient-centered ambulatory care in interventional endoscopy, paving the way for future, more explicit AI-enhanced ambulatory surgery protocols.

**Figure 3:**
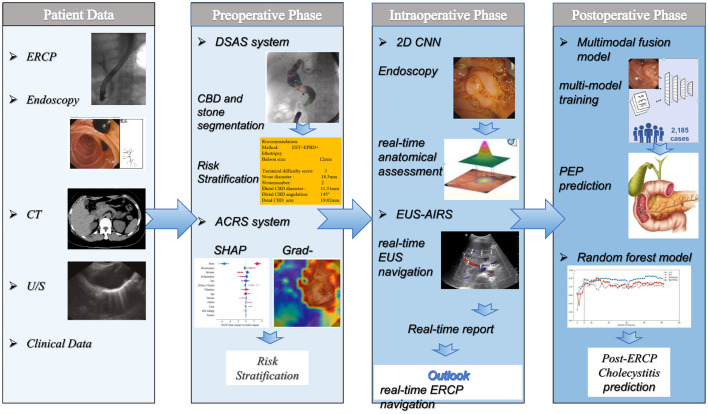
Integrated AI Clinical Workflow for ERCP. This figure depicts the seamless AI-enhanced workflow encompassing patient data collection, preoperative risk stratification using multimodal inputs, intraoperative real-time navigation and decision support, and postoperative complication prediction. Arrows indicate the sequential clinical pathway, forming a closed-loop AI management system for comprehensive ERCP care. ACRS, assessment of stone removal challenges; AI, artificial intelligence; CBD, common bile duct; CNNs, convolutional neural networks; CT, computed tomography; DSAS, difficulty scoring system; EUS-AIRS, automatic image reporting system; ERCP, endoscopic retrograde cholangiopancreatography; Grad-CAM, gradient-weighted class activation mapping; PEP, post-endoscopic retrograde cholangiopancreatography pancreatitis; SHAP, shapley additive explanations; US, ultrasound.

Despite the demonstrated potential of AI in optimizing perioperative ERCP workflows, a systematic synthesis evaluating model performance, clinical applicability, and inherent limitations remains imperative. Table 1 (ERCP-Specific AI Models: Technical Metrics Across Procedural Phases) comprehensively summarizes representative AI technologies across preoperative, intraoperative, postoperative risk prediction, and integrated perioperative management domains. Complemented by Figure 3, which illustrates clinical integration pathways, this multi-component analysis provides evidence-based guidance for technology selection and iterative refinement.

**Table 1:** ERCP-specific AI models: technical metrics across procedural phases.

Application field	Author/Team	Algorithm type	Sample size	Validation method	Application scenarios	Accuracy (%)	Sensitivity/ Specificity (%)	PPV / NPV (%)	AUC (95% CI)	F1 score
Preoperative risk assessment	Hou et al. (12)	EfficientNet-B5 (CNN)	789 patients, 3,156 images	Multicenter retrospective	High-risk patient screening for bile duct dilation >10mm	91.68	91.16 / 93.25	NA	0.95 (0.93–0.97)	0.92
	Huang et al. (13)	U-Net++	3 hospitals, 1,954 images	Prospective multicenter trial	Bile duct stone extraction difficulty scoring	NA	NA	NA	NA	NA
	Wang et al. (15)	DeiT + XGBoost	2,129 patients	Internal validation	Personalized surgical planning for stone removal	83	100 / 77	85 / 100	0.89 (0.86–0.92)	0.92
Intraoperative navigation	Kim et al. (19)	2D CNN	982 patients	Single-center retrospective	Real-time ampulla localization and cannulation difficulty	76.2	NA	NA	0.81 (0.78–0.84)	0.74
	Li et al. (23)	3D spatiotemporal network (EUS-AIRS)	114 patients	Retrospective + prospective trial	Biliary-pancreatic anatomical recognition and reporting	90.8	NA	NA	0.94 (0.91–0.97)	0.88
Postoperative complication prediction	Meng et al. (26)	Logistic regression	4 hospitals, 3,021 patients	Multicenter retrospective cohort	PEP risk prediction	79	NA	78 / 80	0.82 (0.79–0.85)	0.79
	Xu et al. (28)	Multimodal fusion model	1,916 patients	Dynamic risk assessment (multicenter)	PEP prevention	89	83.33 / 85.92	84 / 87	0.91 (0.88–0.94)	0.86
	Zhang et al. (33)	Random forest	1,117 patients	Single-center retrospective	Post-ERCP cholecystitis risk prediction	85.5	82.2 / 85.3	NA	0.890 (0.86–0.92)	NA
Anesthesia Management	Biro et al. (36)	REALITI (robotic vision)	Human model trials	Simulator validation	AI-assisted endotracheal intubation using laryngeal landmarks	95	NA	NA	0.97 (0.95–0.99)	0.96

This table summarizes key specifications and performance metrics for representative AI algorithms applied across the ERCP workflow, encompassing preoperative risk assessment, intraoperative navigation, postoperative complication prediction, and anesthesia management. AI, artificial intelligence; CNN, convolutional neural network; DeiT, data-efficient image transformers; ERCP, endoscopic retrograde cholangiopancreatography; EUS-AIRS, endoscopic ultrasonography automatic image reporting system; PEP, post-endoscopic retrograde cholangiopancreatography pancreatitis; REALITI, robotic endoscope-automated via laryngeal imaging for tracheal intubation; XGBoost, eXtreme gradient boosting; PV, positive predictive value; NPV, negative predictive value; F1, F1 score (harmonic mean of precision and recall); AUC, area under the curve; CI, Confidence interval; NA, not available.

## 5 Future research directions

### 5.1 Multicenter collaboration, multimodal AI integration, and data standardization

The establishment of standardized multicenter databases and multimodal AI systems is critical to address clinical heterogeneity and feature drift in AI-assisted ERCP applications. Unimodal data analysis is insufficient to capture the complexity of patient presentations, largely due to variations in imaging protocols, device parameters, and inconsistent annotation practices—all of which impair model generalizability (44). Harmonized databases—using normalization techniques such as the Combat model (validated across 36 centers for MRI data)—enable device-independent parameter adjustments and adaptive transformations, thereby reducing technical heterogeneity while preserving biologically relevant features (45, 46). Multimodal AI approaches [e.g., MuMo (47, 48), which integrates endoscopic images, clinical notes, and laboratory results] can improve predictive performance by 18–23% over unimodal models by simulating human clinical reasoning. Future efforts should prioritize the creation of interoperable data pipelines, ensure computational scalability, and maintain rigorous regulatory compliance to facilitate the transition of AI from experimental prototypes to clinically validated platforms (49).

### 5.2 Ethical, legal, and policy frameworks for medical AI

The rapid adoption of robotic-assisted surgery has highlighted several limitations in existing medical liability frameworks, particularly in the context of the “black-box” nature of AI decision-making processes. For instance, CNN models in ERCP have achieved lesion detection accuracies as high as 92.3%, yet their decision-making processes remain largely opaque (50). Current practice allows for some degree of interpretability through transparent technical explanations. In addition, the disclosure of algorithm training datasets and known limitations via model cards—akin to pharmaceutical package inserts—can help physicians and patients make informed decisions (51). Finally, AI tools such as ChatGPT may further support the generation of informed consent documents and facilitate patient education (52, 53).

When AI contributes to misdiagnosis, responsibility may fall on the physician, the healthcare institution, or the algorithm developer. However, current domestic regulations reveal significant gaps in assigning liability (54). The EU AI Act offers a reference model, stipulating that the deployer (physician or hospital) bears ultimate responsibility and must prove that AI tools were used in accordance with established protocols (55). Similarly, the ESGE Guidelines (2022) emphasize that physicians must verify the clinical appropriateness of AI recommendations; failure to do so constitutes negligence (56). Future policy frameworks should adopt tiered liability models and two-phase approval systems—Phase 1 to assess safety and generalization, and Phase 2 to confirm evidence-based clinical validity via multicenter trials (57, 58).

### 5.3 AI-enabled comprehensive patient journey management: safety, economics, and communication

Beyond technical performance, the successful adoption of AI depends on its ability to optimize real-world healthcare economics, ensure patient safety, and strengthen communication throughout the care continuum. Cost-effectiveness can be enhanced through lightweight AI interfaces [e.g., Fast Healthcare Interoperability Resource (FHIR) standards] for seamless HER (Electronic Health Records) integration, alongside targeted training in human-AI collaboration to improve user confidence (59, 60). Perioperative workflows may benefit from ML-driven risk-stratified scheduling systems that adapt appointment times to case complexity (61), and closed-loop systems for 24-h ambulatory ERCP. Postoperatively, Natural Language Processing (NLP) tools such as ChatGPT can automate the generation of personalized rehabilitation plans and patient education materials (52, 53, 62), while dynamic complication prediction models (e.g., PEP risk recalibration) can guide preemptive interventions. Future research should focus on AI-driven preoperative education, real-time cost-benefit analytics, and integrated communication platforms to unify the patient journey from admission to recovery.

### 5.4 Staged implementation roadmap for AI-assisted ERCP

The integration of artificial intelligence into ERCP clinical practice necessitates a structured, evidence-based, and phased implementation strategy. A cautious, stepwise approach—progressing from low-risk assistance to higher-complexity tasks—ensures patient safety, facilitates regulatory compliance, and builds clinical trust. Table 2 outlines three distinct phases, specifying key objectives, representative AI functions, evidence requirements, and anticipated regulatory hurdles for each stage.

**Table 2:** Phased roadmap for clinical integration of AI in ERCP.

Phase & timeline	Core focus	Example AI functions	Key evidence required	Major barriers
PhaseI (1-2 years) Low-Risk Assistance	Workflow augmentation and data standardization.	Automated reporting, image standardization, and basic landmark ID.	Efficiency & usability studies; data interoperability proof.	IT integration; data security; SaMD classification.
Phase II (3-5 years) Medium-Risk Diagnostic Support	Diagnostic and planning support (human-in-the-loop).	Stone/stricture detection, risk stratification, and real-time navigation.	Prospective accuracy & multicenter outcome studies.	Class II approval; liability frameworks; reimbursement.
Phase III (5+ years) High-Risk Autonomous Functions	Supervised autonomous guidance for sub-tasks.	Semi-autonomous cannulation, dynamic complication prevention.	RCTs for safety/efficacy; long-term surveillance; health economics.	Class III approval; liability for autonomy; ethical acceptance.

This roadmap proposes a stepwise, evidence-driven approach for integrating AI into ERCP. Progression between phases depends on accumulating robust clinical validation and addressing escalating regulatory, liability, and infrastructural challenges. Class II (moderate-risk) and Class III (high-risk) refer to regulatory classifications for software as a medical device (SaMD), requiring increasing levels of clinical evidence for approval. AI, artificial intelligence; ERCP, endoscopic retrograde cholangiopancreatography; ID, identification; PEP, post-endoscopic retrograde cholangiopancreatography pancreatitis; RCT, randomized controlled trial; SaMD, software as a medical device; XAI, explainable artificial intelligence.

### 5.5 Barriers to widespread clinical adoption and proposed solutions

Despite promising results, several obstacles impede routine AI deployment in ERCP. Table 3 summarizes the major barriers across four domains and offers pragmatic countermeasures.

**Table 3:** Barriers to widespread clinical adoption and proposed solutions.

Barrier domain	Specific barriers	Proposed solutions
Technical & data	•Scarce annotated ERCP imaging datasets •Domain shift across endoscopy systems •Real-time inference latency	•Establish multicenter imaging repositories with standardised annotation (ERCP-AI consortium) •Apply domain adaptation (adversarial training, normalisation) •Deploy edge-optimised inference
Clinical workflow	•Disruption of existing reporting routines •Poor interoperability with EHR/endoscopy suites	•Build FHIR-based AI modules that plug into current systems (EndoWorks, Olympus EndoAlpha) •Introduce AI as a silent second reader initially
Regulatory & legal	•No ERCP-specific AI approvals •Unclear liability for AI-influenced decisions	•Pursue de novo FDA/CE clearance for low-risk tasks (e.g., image enhancement) •Establish local AI oversight committees •Mandate human-in-the-loop for high-risk decisions
Human factors	•Clinician mistrust of “black-box” models •Inadequate training in AI interpretation	•Provide explainability (Grad-CAM, SHAP) by default •Develop CME-accredited courses on AI-assisted endoscopy •Engage clinical champions for peer-to-peer education

This table presents a structured framework for categorising the major barriers that currently limit routine clinical adoption of AI-assisted ERCP, alongside actionable, domain-specific solutions for each category. Addressing these obstacles—spanning technical/data limitations, clinical workflow disruptions, regulatory/legal uncertainties, and human factors—requires a multi-layered, phase-aware strategy that combines short-term fixes (e.g., data harmonisation and silent-mode clinical integration) with long-term structural reforms (e.g., regulatory clearance pathways and formalised training curricula). Distinguishing between intrinsic model constraints (data availability, computational latency) and extrinsic implementation barriers (interoperability, liability, user trust) is critical for prioritizing interventions. AI, artificial intelligence; ERCP, endoscopic retrograde cholangiopancreatography; EHR, electronic health record; FDA, U.S. food and drug administration; FHIR, fast healthcare interoperability resources; XAI, explainable artificial intelligence.

## 6 Conclusion

AI demonstrates significant potential for transforming ERCP by enhancing precision and safety across the entire clinical workflow. Its potential applications span preoperative planning through predictive analytics, intraoperative navigation via computer vision, and post-procedural monitoring with real-time complication detection—ultimately aiming to improve pancreatobiliary management. However, many AI systems remain at an early development stage or are limited to small-scale pilot testing. Implementation faces substantial challenges, including the lack of data standardization, ethical concerns regarding algorithmic autonomy, limited validation across diverse clinical settings, and the need for robust digital infrastructure. Addressing these barriers will require multidisciplinary collaboration to develop explainable AI systems, harmonized data-sharing protocols, supportive regulatory frameworks, and rigorous real-world validation. As these solutions mature and undergo extensive multicenter testing, AI could evolve from a promising adjunct into a fully integrated decision-support system, setting new standards for personalized pancreatobiliary care while maintaining essential clinician oversight.
